# A multistage model for rapid identification of geological features in shield tunnelling

**DOI:** 10.1038/s41598-023-28243-6

**Published:** 2023-01-31

**Authors:** Min Hu, Jing Lu, WenBo Zhou, Wei Xu, ZhaoYu Wu

**Affiliations:** 1 SHU-SUCG Research Centre for Building Industrialization, Shanghai University, Shanghai, 200072 China; 2 SHU-UTS SILC Business School, Shanghai University, Shanghai, 201800 China; 3 School of Mechanical and Electrical Engineering and Automation, Shanghai University, Shanghai, 200072 China; 4 Shanghai Tunnel Engineering Co., Ltd., Shanghai, 200232 China

**Keywords:** Civil engineering, Scientific data

## Abstract

Decision-making on shield construction parameters depends on timely and accurate geological condition feedback. Real-time mastering of geological condition around the shield during tunnelling is necessary to achieve safe and efficient construction. This paper proposes a Rapidly Geological Features Identification (RGFI) method that balances the model's generalizability and the accuracy of geological identification. First, a k-means algorithm is used to redefine the stratum based on the key mechanical indexes of strata. An XGBoost model is then used to determine the stratum composition of the excavation face based on the tunnelling parameters. If the result is compound strata, a deep neural network with an attention mechanism is used to predict the percentage of each stratum. The attention mechanism assigns weights to the features of the tunnelling parameters according to the stratum composition. The simulation results in the interval between Qian-Zhuang and Ke-Ning Road of Nanjing Metro show that the method can effectively determine the geological conditions on the excavation face. Furthermore, the method was used in the Hangzhou-Shaoxing intercity railroad tunnel project, where the 'ZhiYu' self-driving shield was used for tunnelling control. It helped the 'ZhiYu' shield to adjust the construction parameters quickly and improve the safety and quality of the project.

## Introduction

**Figure 1:**
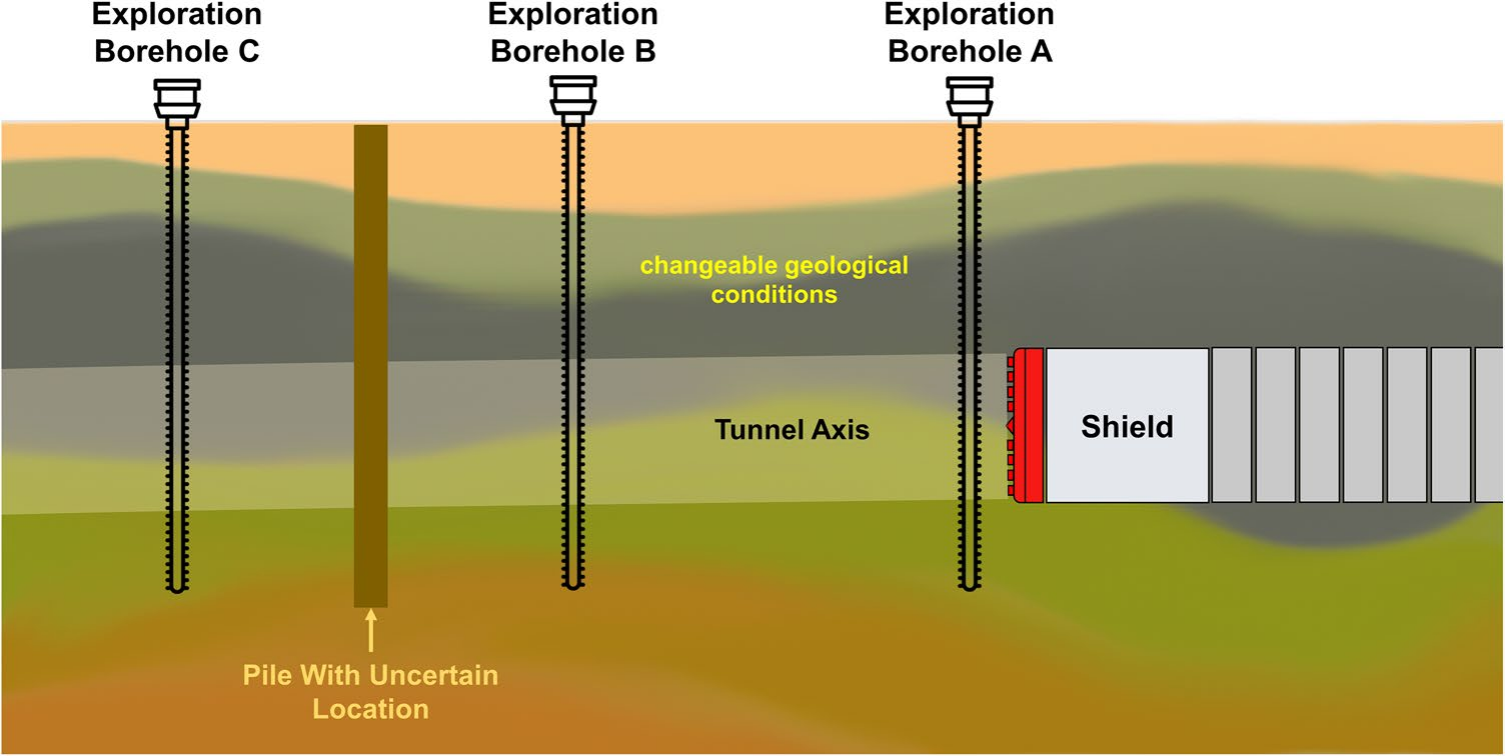
Schematic diagram of shield tunnelling.

With the rapid development of artificial intelligence technology, intelligent decision-making in shield tunnelling has become hot research in tunnel construction^1^. The shield machine and the geotechnical medium interact with each other^2^. Due to the unpredictable and highly complex geological environment during tunnelling, once the control parameters are set to mismatch with the surrounding geology, it will affect the tunnelling performance, cause disturbance to the surrounding soil, and even cause serious engineering accidents^3^. Geological exploration is conducted before tunnel construction to grasp the geological information of the construction area. In geological exploration, geological boreholes are usually carried out along the axis of tunnel design at specific intervals to obtain rock and soil samples for detection. The investigators estimate geological conditions between adjacent geological boreholes based on experience, but geological conditions obtained by geological exploration may not be accurate. Due to the constraints of the survey conditions, some boreholes were off the tunnel axis, or the distance between adjacent boreholes was too far. Therefore, the accuracy of the geological information obtained based on geological exploration is not high because the properties of the soil not directly sampled and tested may differ from those of the nearby boreholes, and some construction waste remains underground^12^ (as shown in Fig. 1). Therefore, it is necessary to find a method to precisely identify the geological condition of the excavation face based on geological exploration to help the shield machine quickly identify the geological condition and adjust the tunnelling control strategies timely. Methods to obtain the geological conditions in front of the excavation during the tunnelling process can be divided into advanced geological forecasting methods and geological identification methods based on tunnelling data. The sensor-based geological forecasting methods specifically include the seismic wave method, electric method, and horizontal sound probing method. The seismic wave method^4,5^ predicts geological conditions by analyzing the reflected wave data by hammering the front soil layer during shield shutdown to generate seismic waves. The electrical method^6,7^ predicts the integrity and water content of the surrounding rock in front by exciting an electric field in the subsurface geotechnical body and detecting the frequency effect and the change of apparent resistivity in front. The horizontal sound probing method^8^ uses the signal excited by cutting the rock for adverse geological body forecasting. These methods can effectively predict geological conditions to a certain extent. However, the complex magnetic field environment interferes with the electronic equipment because of the small space inside the shield machine. The existing geological forecasting techniques have limited accuracy and high cost. Therefore, these methods are mainly used in specific geological areas such as karst.

Because of big data technology, real-time tunnelling data for shield tunnel construction are used to establish geological identification models. These methods identify the geological condition of the excavation face by modeling the relationship between the tunnelling data and the geological types. Regression analysis, statistical methods, and machine learning are the most used algorithms. Nelson et al.^9^ used a linear regression method, and Hassanpour et al.^10^ used a logarithmic regression method to find the relationship between tunnelling parameters and rock parameters at the excavation face. It achieved good results in specific projects. Leu et al.^11^ classified the geological risk of the excavation face into three states and proposed a probabilistic model of tunnel risk based on a real-time Bayesian analysis method. Guan et al.^12^ determined the correspondence between the geological parameter combination and the grade of ground conditions by establishing a geological table and then calculated the probability distribution of each geological parameter between the excavation face and known geological boreholes using the Markov random process and the Bayesian method. The geological classification method based on statistics is characterized by high interpretability and fast operation, which allows a simple classification of geological types^13,14^.

With machine learning techniques showing good performance in solving nonlinear problems in recent years, they have been increasingly used in shield geological identification^15,16^. Shield tunnelling data are the input of such identification models, and geological types or geological parameters are the output, but the definitions of geological type and the level of refinement of category division vary. Most researchers directly train their models using the geological types in the geological exploration results as labels. For example, Zhang et al.^17^ used a chi-square test to screen out the seven tunnelling parameters most sensitive to geological changes and constructed a geological-type prediction model using a classification and regression tree algorithm based on data from geological exploration. Shi et al.^18^ used a deep neural network (DNN) model to predict seven geological types of excavation faces using 53 shield excavation parameters as input. Liu et al.^19^ considered the spatio-temporal features of the tunnelling data and used a long short-term memory network based on the global attention mechanism to improve the accuracy of geological type classification effectively. The existing stratum classification has different standards in different areas, and the model accuracy judgment standard is based entirely on geological exploration reports. Therefore, although the accuracy of these models is high, the model generalization capability needs to be improved. Geological condition identification was used to overcome these limitations and estimate geological parameters and characteristics. Liu et al.^20,21^ found a rock mass prediction model based on shield tunnelling parameters using the backpropagation neural network and the improved support vector regression model. These approaches predicted rock mass parameters, including uniaxial compression strength, brittleness index, the distance between planes of weakness, and the orientation of discontinuities. The model is suitable for predicting geological parameters in the case of known geological types. Some researchers reclassify the geological type of the excavation face. Jung et al.^22^ classified the excavation face into three types based on the proportion of soft and hard stratum and predicted the geological type in front of the shield using tunnelling parameters such as advance speed, thrust force, and cutterhead torque. This classification method has good generalization but low precision. Sebbeh-Newton et al.^23^ used the JH system^24^ to classify the rock mass into four categories and established a prediction model based on the random forest method. Zhang et al.^25^ used the K-means algorithm to find potential rock mass types in the shield tunnelling data and predicted the geological conditions using a support vector classifier model. Yan et al.^26,27^ integrate grid search (GS) and K-fold cross-validation (K-CV) into the overlay classification algorithm (SCA) according to the data generated by shield tunnelling and borehole data to realize the prediction of geological characteristics.

In summary, the sensor-based geological forecasting method can realize geological forecasting. However, because of its limited accuracy and high cost, it is mainly applied to detect adverse geology such as karst. The current geological identification method based on machine learning has low cost and high real-time performance. However, the results of these prediction models are not sufficiently precise and are dominated by major geological types, lacking a description of the refined stratum types and distribution of strata at the cutting face. In addition, the traditional stratum types are difficult to provide sufficient information for adjusting shield excavation parameters because of the weak correlation between geological classification and shield excavation characteristics. Therefore, a construction-oriented geological condition identification method needs to be investigated.

This paper introduces construction-oriented stratum distribution characteristics research based on soil mechanical indexes and shield tunnelling parameters. A three-stage Rapidly Geological Features Identification model (RGFI) is proposed, which includes stratum redefinition, geological type classification, and stratum distribution prediction. First, this method redefines the stratum based on key mechanical indicators to improve model generalization and solve the problem of regional differences in stratigraphic classification standards. Then an XGBoost model is used to determine the stratum composition of the excavation face based on the tunnelling parameters. Finally, a deep neural network with an attention mechanism is used to predict the percentage of each stratum. The attention mechanism assigns weights to the features of the tunnelling parameters according to the stratum composition.

This paper comprises five sections. "Methods" introduces the framework and algorithm flow of the RGFI. "Engineering simulation and analysis" presents the simulation results of the method on the Nanjing Metro Line 5 project and conducts a comparative analysis of different models. "Engineering applications" introduces the application of the RGFI method in the Hangzhou-Shaoxing grade railway tunnel project. Finally, "Conclusions" concludes the whole paper.

## Methods

### Framework

The RGFI is based on the existing geological exploration information and uses real-time tunnelling data of the shield machine to identify and correct the geological information in front of the excavation face to provide more accurate geological information for the shield machine to make autonomous decisions.

**Figure 2:**
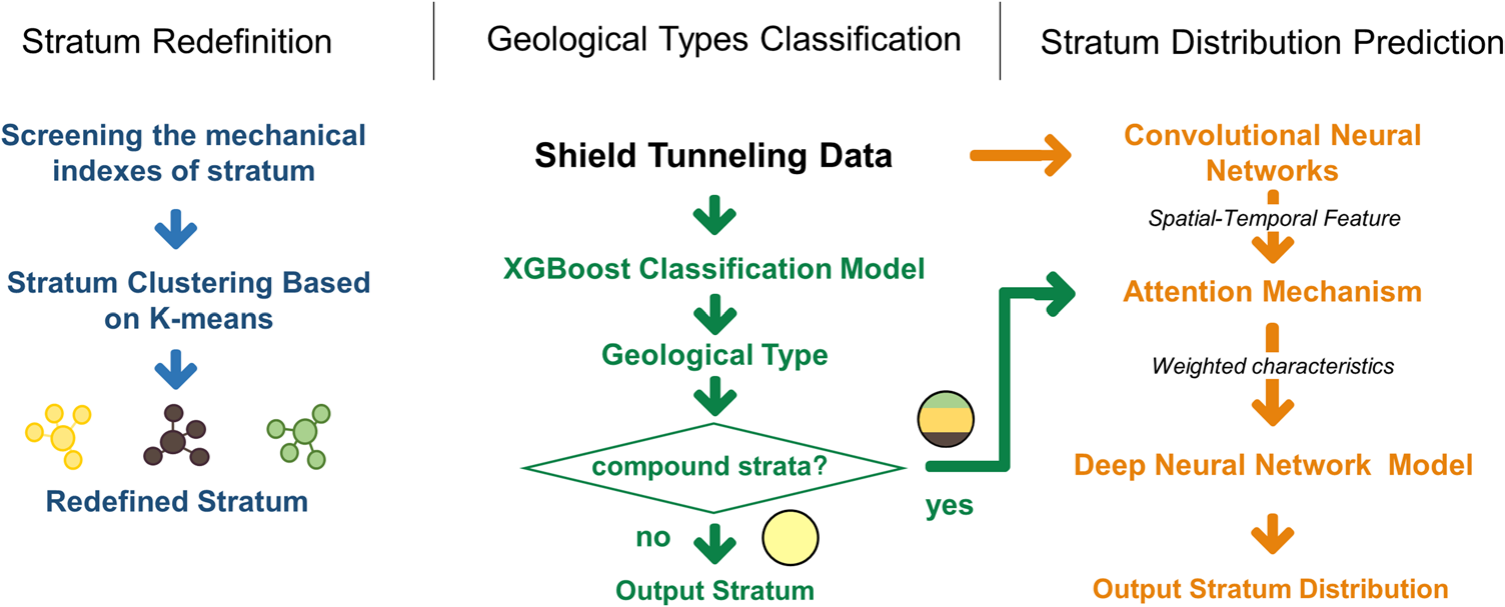
Schematic diagram of rapidly geological features identification model.

The method involves three steps, as shown in Fig. 2: (1) Stratum redefinition. Geotechnical parameters highly correlated with shield tunnelling performance are selected from the geological exploration report, and k-means clustering is used to redefine the stratum type. (2) Geological type classification. Using the feature engineering method, the highly geotype-dependent shield tunnelling parameters were selected. Then, the XGBoost geological type classification model is trained to achieve real-time geological type identification. (3) Stratum distribution prediction. A convolutional neural network is used to extract the Spatio-temporal features of shield excavation data. Then, the attention mechanism is used to assign weights to the extracted features in combination with real-time geological type classification results. Finally, the percentage of stratum in the excavation face is obtained using a deep neural network.

### Feature engineering

There are lots of shield tunnelling parameters, and not all parameters can reflect the changes in geological conditions. Therefore, it is necessary to filter the shield tunnelling parameters before developing the model. In this paper, Maximal Information Coefficient (MIC) was used to measure the relevance between two variables, and because it can discover the nonlinear and potential nonfunctional correlations in data sets^28^. Suppose the data of a tunnelling parameter is $u=\left\{{u}_{i}|i=1,...,k\right\}$, and the geological types are $g=\left\{{g}_{i}|i=1,...,k\right\}$. $R=\left\{\left({u}_{i},{\mathrm{g}}_{i}\right),i=1,2...,k\right\}$ is a finite set of ordered pairs. The grid $\mathrm{D}$ divides the domain of values $u$ and $g$ into $m$ and $n$ segments; then, $m*n$ grid partitioning is obtained. The MIC values of $u$ and $g$ are calculated as shown in Eqs. (1–3)^28^:

1 $$MI\left({X}^{*},{Y}^{*}\right)=\sum_{{x}^{*}\in {X}^{*}}\sum_{{y}^{*}\in {Y}^{*}}p\left({x}^{*},{y}^{*}\right)\mathrm{log}\frac{p\left({x}^{*},{y}^{*}\right)}{p\left({x}^{*}\right)p\left({y}^{*}\right)},$$

where $MI({X}^{*},{Y}^{*})$ is the mutual information of ${X}^{*}$ and ${Y}^{*}$, $p({x}^{*},{y}^{*})$ is the joint probability density, and $p({x}^{*})$ and $p({y}^{*})$ are the marginal probability density.

Then, the maximum mutual information $M{I}^{*}(R,u,g)$ of $R$ on the grid $\mathrm{D}$ is calculated as follows:

2 $$ MI^{*} \left( {R,u,g} \right) = \max MI\left( {{\text{R}}|{\text{D}}} \right). $$

The maximum mutual information coefficient between the tunnelling parameters ${x}_{j}$ and the geological types $y$ is given by

3 $$MIC\left(u,g\right)=\underset{m*n<B}{\mathrm{max}}\frac{M{I}^{*}(R,u,g)}{\mathrm{logmin}\left\{u,g\right\}},$$

where $\mathrm{B}$ is the upper limit of the grid division result $m*n$; usually, $\mathrm{B}={k}^{0.6}$, where $k$ is the number of data samples.

We performed preliminary screening of the original shield tunnelling parameters based on prior studies^17,29,30^ and removed parameters that were not relevant for geological condition determination. According to the studies of Jung et al.^22^ and Song et al.^29^, we added three comprehensive indicators to the original data set: penetration rate ($PR$), the ratio of advance speed to total thrust ($ATR$), and the ratio of cutterhead torque to cutterhead rotational speed ($TSR$). The added indicators are calculated as follows^22,29^:

4 $$PR=\frac{Ar}{Rpm},$$

5 $$ATR=\frac{Ar}{Th},$$

6 $$TSR=\frac{Tr}{Rpm},$$

where $Ar$ is the advance speed, $Rpm$ is the cutterhead rotational speed, $Tr$ is the cutterhead torque, and $Th$ is the total thrust.

We calculate the MIC values of each parameter based on history shield tunnel project data, and 14 tunnelling parameters with MIC values greater than 0.5 were used for geological type classification model training. The result is shown in Table 1.

**Table 1:** Results of correlation calculation and selection of shield tunnelling parameters.

Serial number	Parameter	MIC value	Select	Serial number	Parameter	MIC value	Select
1	Total thrust	0.3	False	12	Advance speed	0.68	True
2	Cutterhead rotational speed	0.57	True	13	Total pressure of thrust cylinders	0.67	True
3	Cutterhead oil pressure	0.42	False	14	Thrust cylinders pressure of upper zone	0.53	True
4	Cutterhead torque	0.44	False	15	Thrust cylinders pressure of lower zone	0.51	True
5	Screw conveyor torque	0.46	False	16	Thrust cylinders pressure of left zone	0.58	True
6	Screw conveyor rotational speed	0.59	True	17	Thrust cylinders pressure of right zone	0.68	True
7	Upper chamber earth pressure	0.63	True	18	Thrust of articulation cylinders	0.21	False
8	Lower chamber earth pressure	0.75	True	19	Slope angle	0.34	False
9	Central chamber earth pressure	0.92	True	20	PR	0.72	True
10	Upper screw conveyor pressure	0.34	False	21	TSR	1	True
11	Lower screw conveyor pressure	0.31	False	22	ATR	0.6	True

*ATR* ratio of advance rate to total thrust, *TSR* the ratio of torque to cutter speed, *PR* penetration rate.

The propulsion system, cutter system, and screw machine of the shield are hydraulic actuators. The hydraulic opening and oil pressure relationship will change when the environment changes^31^. Considering the stratum distribution prediction needs more information, we added the set values of operational parameters to the inputs of the model. The stratum distribution prediction input parameters are shown in Table 2.

**Table 2:** Input parameters of stratum distribution prediction model.

System type	Feature name
Propulsion system	Total pressure of thrust cylinders, thrust cylinder pressure in the upper zone, thrust cylinder pressure in the lower zone, thrust cylinder pressure in the left zone, thrust cylinder pressure in the right zone, thrust cylinders valve opening in the upper zone, thrust cylinder valve opening in the lower zone, thrust cylinder valve opening in the left zone, thrust cylinder valve opening in the right zone, advance speed, advance speed setting value, ATR
Cutting system	Cutterhead rotational speed, cutterhead rotational speed setting value, TSR, PR
Soil pressure balancing system	Screw conveyor rotational speed, screw conveyor rotational speed setting value, upper chamber earth pressure, lower chamber earth pressure, central chamber earth pressure

*ATR* ratio of advance rate to total thrust, *TSR* ratio of torque to cutter speed, *PR* penetration rate.

### Stratum redefinition

Stratum divisions in geological exploration are mainly based on geochronology, particle composition, and plasticity index. The rules of stratum numbering vary from region to region. Furthermore, the existing division criteria are not specific to shield construction, and the results are difficult to use directly to guide construction. Therefore, this study proposes a stratum redefinition method for shield construction to re-divide the stratum before performing geological type identification.

In shield tunnelling, the compression modulus, Poisson's ratio, cohesion, internal friction angle, lateral pressure coefficient, and standard penetration value are the most influential geotechnical parameters^32–34^. So we redefine strata based on these parameters.

The K-means algorithm is an unsupervised clustering algorithm that aims to group similar samples in one group and separate dissimilar samples in different groups^35^. The algorithm starts by randomly choosing K initial centroids, where a cluster centroid is the mean of all data points within a cluster. Afterward, each sample is assigned to the nearest center by minimizing the distance between samples and the corresponding centroids. After the clusters are formed, the algorithm iterates this process until no point changes clusters. The distance between samples and the corresponding centroids is calculated using the sum of squared errors ($SSE$)^35^, which is calculated as follows:

7 $$SSE=\sum_{i=1}^{K}\sum_{{p}_{geo}\in {C}_{i}}dist{\left({c}_{i}-{p}_{geo}\right)}^{2},$$

where $K$ is the number of geological type clusters, ${C}_{i}$ is the set of points in *i*th cluster, ${c}_{i}$ is the centroid of the cluster ${C}_{i}$ and ${p}_{geo}$ is a vector of geotechnical parameters in the cluster ${C}_{i}$.

Because the number of clusters cannot be determined directly, we use the elbow method^36^ to obtain the optimal number of clusters. The elbow method assumes that the relationship graph of $SSE$ and $K$ is in the shape of an 'elbow', and the point of inflection on the curve is the optimal number of clusters. The degree of the elbow was regarded as the $SSE\mathrm{ratio}$, the smaller the $SSE\mathrm{ratio}$, the greater the elbow degree. Therefore, the optimal number of clusters can be determined by the values of $SSE$ and $SSE\mathrm{ratio}$^37^. $SSE\mathrm{ratio}$ is calculated as follows:

8 $$SSE\mathrm{ratio}=\frac{SS{E}_{K+1}-SS{E}_{K}}{SS{E}_{K}-SS{E}_{K-1}}$$

where $SS{E}_{K}$ is the $SSE$ of the K-means algorithm corresponding to $K$.

### Geological type classification

In this section, the geological type of the excavation face is first determined based on all combinations of the redefined stratum (Tables 3 and 4, for example). Then, we used the eXtreme Gradient Boosting (XGBoost) algorithm to develop a classification model to realize the real-time geological type identification^38^ and used recursive feature elimination methods to optimize the model.

**Table 3:** Physical and mechanical parameters of the strata of the tunnel project from Qian-Zhuang to Ke-Ning Road.

Stratum number	Stratum name	Moisture content	Compression modulus	Cohesive forces	Internal friction angle	Poisson's ratio	Standard penetration value	Formation pressure coefficient
		%	MPa^−1^	kPa	°	–	Hammer	–
②-1b2-3	Silty clay	28.4	5.31	32.6	15	0.32	9.2	0.42
②-1c-d2-3	Sandy silt	30.9	8.49	10.2	27.6	0.3	10.1	0.48
②-2b4	Muddy silty clay	33.7	3.68	14.1	12.7	0.37	4.1	0.42
②-2c-d2-3	Sandy silt	30.3	7.23	8.1	29.3	0.32	10.6	0.59
②-3b2-3	Silty clay	29.1	5.62	28.8	16.3	0.33	7.9	0.46
③-1b1-2	Silty clay	23.6	8.41	49.1	18	0.29	11.6	0.5
③-2b2-3	Silty clay	26.3	5.25	28.4	15.9	0.34	5.7	0.41
③-2c-d2-3	Sandy silt	22.2	8.05	12	26	0.31	13.8	0.51
③-3b1-2	Silty clay	22.7	8.65	48.4	18.9	0.3	12.4	0.45
③-3d-c1-2	Sandy silt	20.2	12.71	2	30	0.3	20.3	0.43
③-4e	Sandy gravel soil	19.4	9.12	3	32	0.31	21.7	0.43
K2c-2	Strong-weathered argillaceous siltstone	–	11	15	35	0.25	45.1	0.45
K2c-3	Medium-weathered argillaceous siltstone	–	25	20	38	0.25	50.8	0.3

**Table 4:** Stratum redefinition results of the tunnel project from Qian-Zhuang to Ke-Ning Road.

Redefined stratum	Ordinary stratum number	Ordinary stratum name
1	K2c-2	Strong-weathered argillaceous siltstone
	K2c-3	Medium-weathered argillaceous siltstone
2	②-1c-d2-3	Sandy silt
	②-2c-d2-3	Sandy silt
	③-2c-d2-3	Sandy silt
	③-3d-c1-2	Sandy silt
	③-4e	Sandy gravel soil
3	②-1b2-3	Silty clay
	②-2b4	Muddy silty clay
	②-3b2-3	Silty clay
	③-2b2-3	Silty clay
	③-3b1-2	Silty clay
	③-1b1-2	Silty clay

#### Geological Type classification based on XGBoost

The geological type classification model of the excavation face uses the XGBoost algorithm, and the construction process^39^ is as follows: Suppose the input dataset is $\left\{\left({U}_{i},{g}_{i}\right),i=1,2,...,k\right\}$, $U$ is the input data of the XGBoost model. The number of classification trees of the model is ${N}_{tree}$. Then, the geological type classification model output $\widehat{g}$ is calculated by Eq. (4)^39^:

9 $$\widehat{g}=\sum_{nt=1}^{{N}_{tree}}{f}_{nt}\left({U}_{i}\right),{f}_{nt}\in F,$$

where ${f}_{nt}(\cdot )$ is a function of the $nt$-th classification tree and $F$ is the set of classification trees.

Training of the geological type classification model by optimizing the objective function composed of two parts: the regularization term and the loss function, which represent the complexity and accuracy of the model. The objective function of the $t$th iteration is as follows^39^:

10 $$\left\{\begin{array}{c}J\left({f}_{t}\right)=\sum_{i=1}^{k}L\left({g}_{i},{\widehat{g}}_{i}^{t-1}+{f}_{t}\left({U}_{i}\right)\right)+\Omega \left({f}_{t}\right)\\ \Omega \left({f}_{t}\right)=\gamma \cdot {N}_{t}+\lambda \frac{1}{2}\sum_{j=1}^{N}{s}_{j}^{2},\end{array}\right.$$

where $L\left(\cdot \right)$ is the loss function, ${\widehat{g}}_{i}^{t-1}$ is the sum of the output values of the previous trees, $\Omega \left(\cdot \right)$ is the penalty term for the model complexity, $\gamma$ is the regularization parameter for the number of leaves, $\lambda$ is the regularization parameter for the leaf weights, $N$ is the number of leaf nodes, and $s$ is the score of leaf nodes.

#### Classification model optimization based on recursive feature elimination algorithm

To improve the efficiency of the geological type classification model, we use the Recursive Feature Elimination (RFE) algorithm in this paper to reduce the number of model input parameters^40^. RFE is a wrapped algorithm to find the optimal feature subset. The main idea is to iteratively build the model, rank the features according to their importance, eliminate the least important feature at a time, calculate the accuracy of the model with the remaining feature subsets, and finally select the optimal feature set. In this paper, we combine the XGBoost model constructed in "Feature engineering" and evaluate the model validity based on the classification accuracy, which calculates as flowing:

11 $$\mathrm{Acc}=\frac{TP}{\left(FP+TP\right)},$$

where $TP$ is the number of correctly classified samples, and $FP$ is the number of incorrectly classified samples.

### Stratum distribution prediction

**Figure 3:**
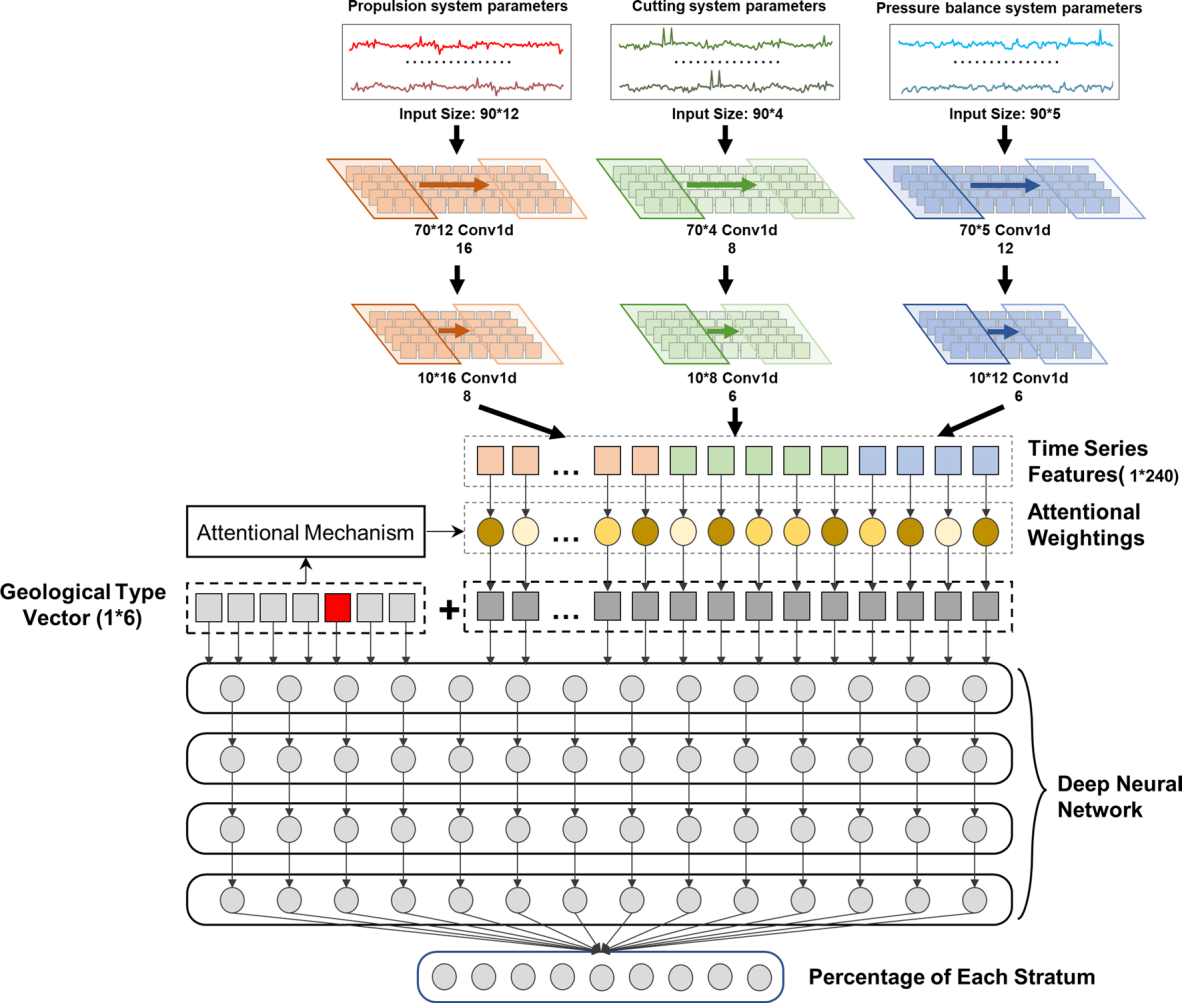
Structure of stratum distribution prediction model.

For a more precise description of the geological conditions in front of the shield, we use one-dimensional convolutional neural networks to deeply mine the Spatio-temporal features of the shield tunnelling data and introduce an attention mechanism to assign different weights to each feature under different geological types that are obtained from the model presented in "Feature engineering". Finally, the weighted spatio-temporal features and geological type classification results are fed into the deep neural network to realize the prediction of the stratum distribution in front of the shield. The model structure diagram is shown in Fig. 3.

#### Spatio-Temporal Features Extraction of Tunnelling Parameter

A one-dimensional convolutional neural network is used to extract the spatio-temporal feature of shield tunnelling data in this study which can directly work on sequence operation with time series data and avoid complicated feature extraction^41^. The model classifies the tunnelling features into a propulsion system, a cutting system, or an earth pressure balance system. The model uses three parallel convolutional neural networks to extract the spatio-temporal features of the tunnelling parameters for separate systems. The feature extraction is accomplished through two one-dimensional convolutional layers, whose convolution kernel width is the same as the input sequence width. The specific operations are shown in Eq. (7)^41^:

12 $${X}_{r}^{l}={\sigma }_{relu}\left(\sum_{v\in {M}_{r}}{X}_{v}^{l-1}{{\omega }_{\mathrm{c}}}_{v,r}^{l}+{{b}_{\mathrm{c}}}_{r}^{l}\right),$$

where ${\sigma }_{relu}\left(\cdot \right)$ is the Relu activation function, ${X}_{r}^{l}$ is the $r$-*th* feature map of the layer $l$, $M$ is the input feature size, ${\omega }_{\mathrm{c}}$ is the weight matrix of the convolution kernel, and ${b}_{\mathrm{c}}$ is the corresponding bias.

In this study, the input data time step is set to 90 to ensure the accuracy and speed of the geological identification model. The low-dimensional spatio-temporal features of tunnelling parameters ${x}_{c}$ are obtained by multiple convolutional transformations with dimensions of 1 × 240, and the compression rate is 12.7%. The number of convolutional kernels for each convolutional layer is shown in Fig. 3.

#### Attention mechanism

The sensitivity of each shield tunnelling parameter is not the same under different geological types^42,43^. Therefore, we use the attention mechanism to assign weight to each spatio-temporal feature according to the geological type to enhance the influence of important features and to attenuate the interference of other features^44^.

The model corrects the spatio-temporal features extracted using a convolutional neural network (CNN) based on the interdependence of the geological type of the excavation face and spatio-temporal features through a layer of neural network modules without bias, which can be expressed as follows:

13 $${w}_{att}={\sigma }_{soft\mathrm{max}}\left(\sum {w}_{a}\widehat{g}\right)$$

where ${\sigma }_{soft\mathrm{max}}\left(\cdot \right)$ is the SoftMax activation function, ${w}_{a}$ is the weight matrix, ${w}_{att}$ is the vector of feature attention weight, and $\widehat{g}$ is the output of the geological type classification model.

Finally, the weighted spatio-temporal feature ${x}_{w}$ is obtained by assigning the corresponding weight to each feature in the spatio-temporal feature ${x}_{c}$ according to ${w}_{att}$.

14 $${x}_{w}={x}_{c}\cdot {w}_{att}.$$

#### Deep neural network

Deep neural networks have powerful nonlinear mapping capabilities and can implement complex regression or classification tasks^45,46^. Therefore, a deep neural network is used for the prediction of stratum distribution. The number of layers of the neural network constructed is four (4), the activation function of layer one (1) to layer three (3) is Relu, and the activation function of layer four (4) is SoftMax. We use the classification results of the geological type and the weighted spatio-temporal feature as the input data of the deep neural network and finally obtain the prediction results of stratum distribution ${y}_{p}$. The calculation process of the model is as follows:

15 $$\left\{\begin{array}{l}x=\left[{x}_{w},\widehat{g}\right]\\ {{h}_{\mathrm{d}}}_{1}={\sigma }_{relu}\left(\sum {{w}_{\mathrm{d}}}_{1}x+{{b}_{\mathrm{d}}}_{1}\right)\\ {{h}_{\mathrm{d}}}_{2}={\sigma }_{relu}\left(\sum {{w}_{\mathrm{d}}}_{2}{{h}_{\mathrm{d}}}_{1}+{{b}_{\mathrm{d}}}_{2}\right)\\ {{h}_{\mathrm{d}}}_{3}={\sigma }_{relu}\left(\sum {{w}_{\mathrm{d}}}_{3}{{h}_{\mathrm{d}}}_{2}+{{b}_{\mathrm{d}}}_{3}\right)\\ {y}_{p}={\sigma }_{softmax}\left(\sum {{w}_{\mathrm{d}}}_{4}{{h}_{\mathrm{d}}}_{3}+{{b}_{\mathrm{d}}}_{4}\right),\end{array}\right.$$

where ${x}_{w}$ is the weighted spatio-temporal feature, ${{w}_{\mathrm{d}}}_{1\sim 4}$ are the weight matrix of each layer, and ${{b}_{\mathrm{d}}}_{1\sim 4}$ are the bias vectors of each layer.

## Engineering simulation and analysis

### Project summary

**Figure 4:**
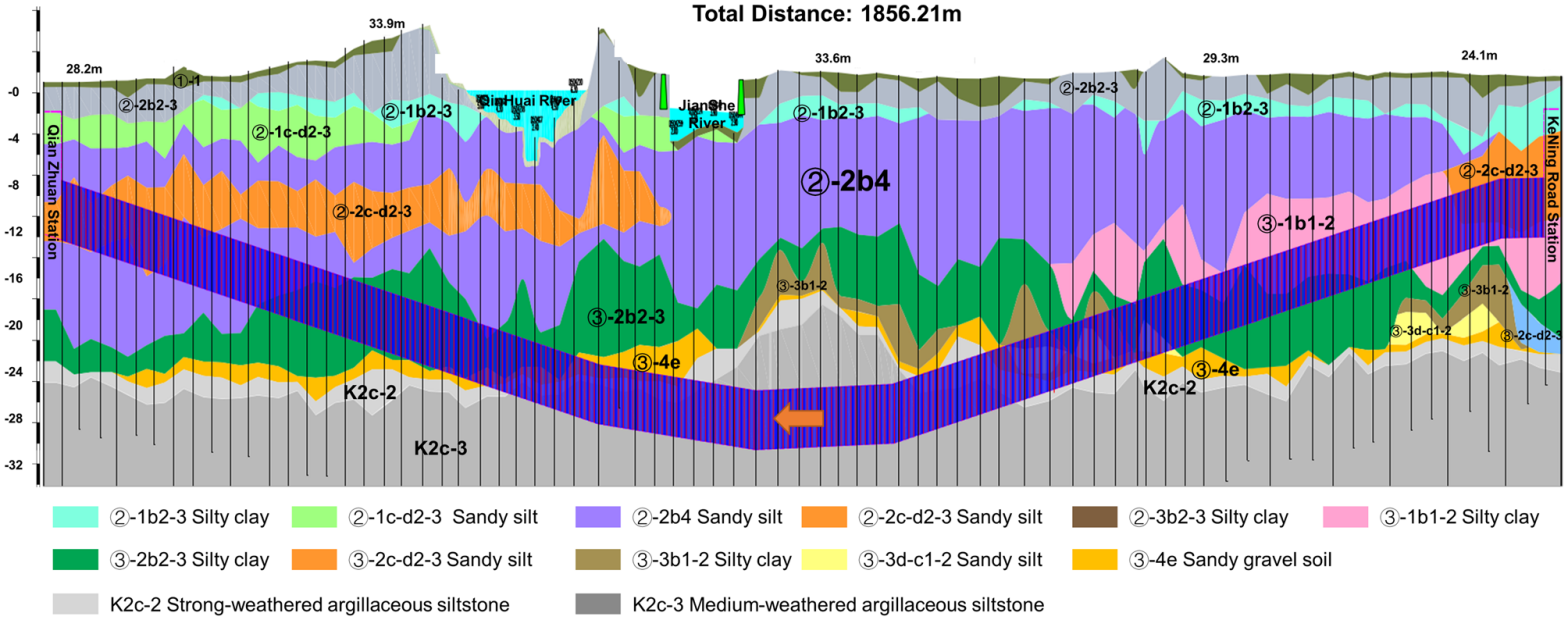
Geological profile of the tunnel project from Qian-Zhuang to Ke-Ning Road.

In this paper, the effect of the RGFI method is analyzed by using the tunnel project of the Qian-Zhuang to Ke-Ning Road interval of Nanjing Metro Line 5 in Nanjing, China, as an experimental project. Nanjing Metro Line 5 was constructed with a 6.46-m-diameter earth pressure balance shield, tunnelling through many types of stratum and complicated geological conditions. Figure 4 shows the geological profile in the geological exploration of the interval from Qian-Zhuang to Ke-Ning Road. The geotechnical parameters are presented in Table 3.

Three types of data are collected: geological exploration, shield tunnelling, and engineering design. The geological exploration data include information on each geological borehole and geotechnical parameters. The shield tunnelling data is collected at a frequency of 1 Hz. The engineering design parameters include the tunnel length, tunnel depth, and the outside diameter and length of the shield machine.

### Data processing

The geological borehole data is sparse compared to the shield tunnelling data. Therefore, to get enough training samples, this paper uses Inverse Distance Weight to calculate the geological data around the boreholes in combination with the cad drawings drawn by the geological technicians.

The original tunnelling data of the shield include a large amount of invalid and abnormal data, which needs to be rejected in advance. These mainly include the data generated during the start-stop phase and after the sensor was interfered with.

To eliminate the adverse effects caused by the different magnitudes of the excavation parameters, we use the z-score standardization method (Eq. 8) that linearly transforms the data to have a mean of zero (0) and a standard deviation of one (1).

16 $${u}_{norm}=\left({u}_{raw}-{u}_{mean}\right)/\sigma ,$$

where ${u}_{raw}$ is the original tunnelling parameter data, ${u}_{norm}$ is the normalized data, ${u}_{mean}$ is the mean value of the data, and $\sigma$ is the variance of the data.

### Stratum redefinition

**Figure 5:**
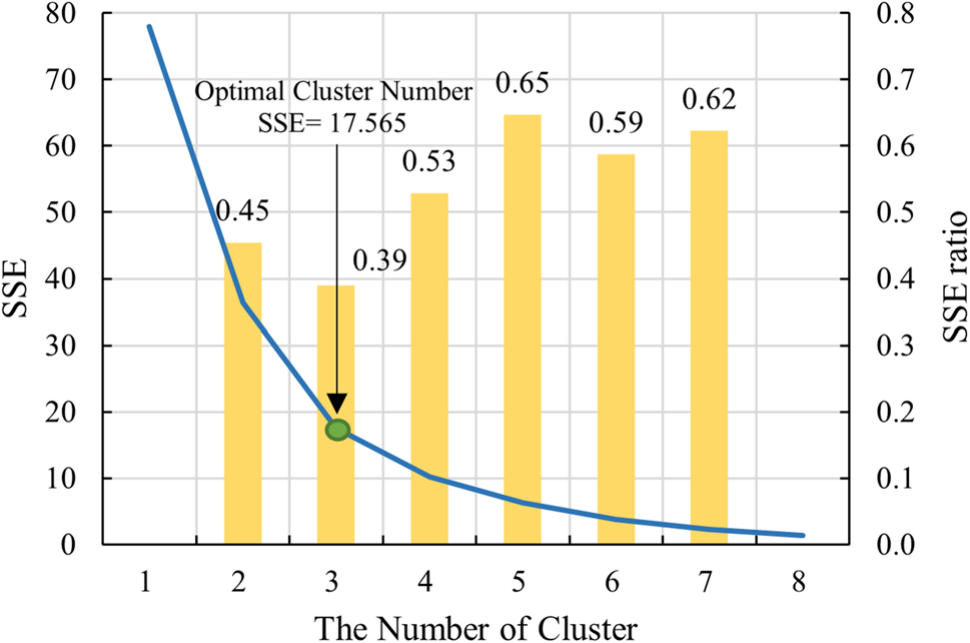
SSE and SSE ratio for different numbers of clusters.

Stratum redefinition was carried out based on the data in Table 3 using the method described in Sect. 2.1, and the results are as follows. Figure 5 shows the change curve of $SSE$ and $SSE\mathrm{ratio}$ with various clusters.

In the graph, the $SSE$ value decreases with increasing $K$. $SSE\mathrm{ratio}$ is minimum at $K=3$. Therefore, the optimal number of clusters was determined to be three (3).

The stratum redefinition results are shown in Table 4. The strata obtained by division are very different from the original stratum number. The original 13 strata are divided into three new strata according to the similarity of the geological parameters related to tunnelling. Stratum 1 consists of argillaceous siltstone with high strength and low compressibility. Stratum 2 consists of silty clay with fine sand interbed and gravel soil, with medium strength and compressibility. Stratum 3 mainly consists of silty clay and gray silt-like soft soil, with low strength and high compressibility.

We identified seven geological types of the excavation face based on possible combinations of stratum types. The results are shown in Table 5. In the process of geological condition identification, the geological type will be identified first, and then the percentage of each stratum will be calculated based on the result.

**Table 5:** Geological type redefinition result of the tunnel project from Qian-Zhuang to Ke-Ning Road.

Geological type	Type 1	Type 2	Type 3	Type 4	Type 5	Type 6	Type 7
Redefined stratum included	1	1, 2	1, 2, 3	1, 3	2	2, 3	3

### Model validation and comparison

**Figure 6:**
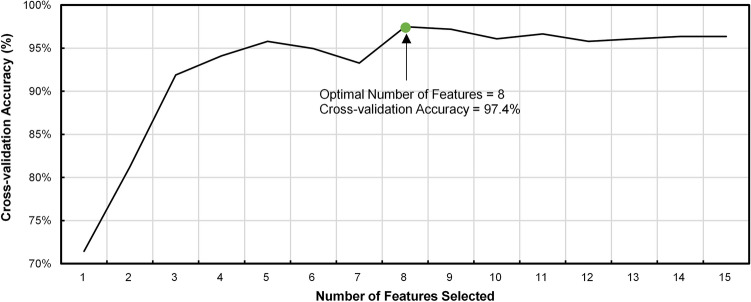
Variation curve of model accuracy with the number of features.

After completing data pre-processing, RGFI was used to identify the geological conditions of the tunnel between Qian-Zhuang and Ke-Ning Road area. Due to the availability of an accurate geological description around the geological borehole, the construction data of 2 m near the borehole point were screened for the training and evaluation of the model. The feature-extracted tunnelling parameters are matched to the geological information by mileage. These data were then randomly divided into a training set, a validation set, and a test set in the ratio of 6:2:2. The geological type classification model was completed with model training and optimization on the training and validation sets. The optimization results based on Recursive Feature Elimination are shown in Fig. 6. The model classification accuracy is the highest when the number of features is eight. The feature sets for stratum classification are optimized as: lower chamber earth pressure, central chamber earth pressure, advance speed, total pressure of thrust cylinders, thrust cylinders pressure of lower zone, PR, TSR, and ATR. The model parameter settings of XGBoost and DNN in the RGFI are presented in Table 6.

**Table 6:** RGFI Model parameters.

XGBoost		DNN	
Parameter	Value	Parameter	Value
Subsample ratio of columns	0.57	Neurons number of the 1st dense	256
Learning rate	0.2	Neurons number of the 2nd dense	256
Max depth	5	Neurons number of the 3rd dense	128
Min child weight	2	Neurons number of the 4th dense	64
n estimators	77	Epochs	2000
Subsample	0.8	Batch size	24

**Figure 7:**
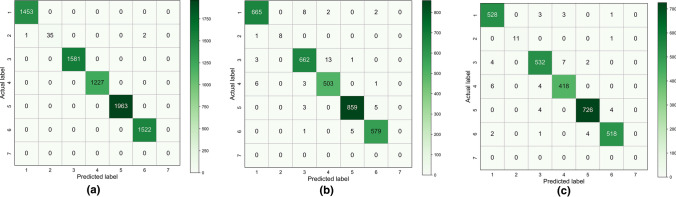
Confusion matrix of the geological type classification model.

Figure 7 shows the confusion matrix of the geological type classification model on the training data, validation data, and test data. Each row of the matrix represents the instances in an actual type while each column represents the instances in a predicted type. We can easily see whether the system is confusing two types. For example, when determining geological type 4 (containing stratum 1, 3) versus geological type 3 (containing stratum 1, 2, 3) on the test set. Geological type 3 was misjudged as geological type 4 seven times, and geological type 4 was misjudged as geological type 3 four times. Most classification errors in the test set occur between similar geological types. The calculated accuracy of the model on the training data, validation data, and test data was 99.96%, 98.37%, and 98.34%, respectively. Table 7 presents the R-squared and root-mean-square errors of the stratum distribution model on different data sets. The results show that the proposed method can effectively determine the precise geological condition of the excavation surface.

**Table 7:** Model performance on different data sets.

	Mean square error	R square
Training set	0.0038	0.9960
Validation set	0.0050	0.9918
Test set	0.0056	0.9863

In this paper, several common data-driven prediction models, including deep neural networks, XGBoost, random forest (RF), and support vector regression (SVR), is selected for comparison experiments with RGFI models for stratum distribution prediction with results shown in Table 8. The RGFI model has a large improvement in accuracy compared with other prediction models, and the mean square error was reduced by 61.9% and 83.9% compared with the random forest model and the deep neural network model, respectively, and the R-square value was also substantially improved, which greatly reduces the model prediction error.

**Table 8:** Performance of different models in stratum distribution prediction.

Model name	R square	MSE
RGFI	0.989	0.00458
RF	0.9431	0.01203
DNN	0.8967	0.02852
XGBoost	0.4199	0.11612
SVR	0.4087	0.09513

## Engineering applications

### Engineering background

**Figure 8:**
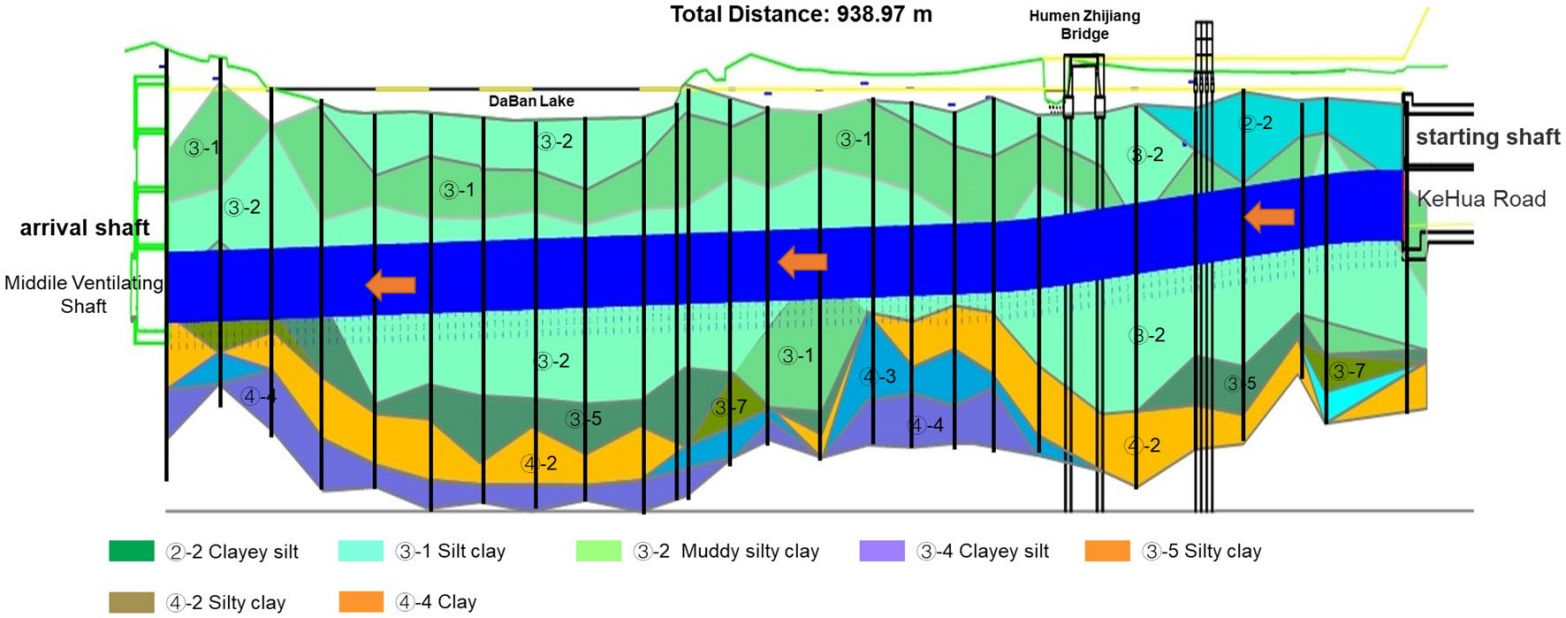
Geological profile of the tunnel project from Kehua Road **to** the middle ventilating shaft.

RGFI was used in the Hangzhou-Shaoxing intercity railroad tunnel project in China, where the 'ZhiYu' self-driving shield was used for tunnelling control. RGFI is an important part of the intelligent control of the 'ZhiYu' shield. With the assistance of RGFI, the 'ZhiYu' shield completed the automatic boring of the left line of the interval between Kehua Road and the middle ventilating shaft, which has a total tunnel length of 938.97 m, a diameter of 6.6 m, and a top burial depth of 10.3–19.7 m. The main soil layers traversed in this tunnel are silt clay, muddy silty clay, silty chalky clay layer, and chalky clay, as shown in Fig. 8. The main geological problems faced during the unmanned tunnelling in this area are the difficulty in controlling the shield attitude due to the change of water content of the stratum and the threat to construction and equipment safety caused by the underground pile legacy with an unknown location. The geotechnical parameters of the stratum crossed by the tunnel in this area are presented in Table 9.

**Table 9:** Geotechnical parameters of the tunnel project from Kehua Road to the middle ventilating shaft.

Stratum number	Stratum name	Moisture content	Compression modulus	Cohesive forces	Internal friction angle	Poisson's ratio	Standard penetration value	Formation pressure coefficient
		%	MPa^−1^	kPa	°	–	Hammer	–
③-1	Silt clay	49.3	2.24	10.4	10.6	0.35	–	0.35
③-2	Muddy silty clay	41.7	2.79	13.2	12.8	0.3	–	0.35
③-4	Clayey silt	32.5	8.59	10.6	27.5	0.3	2.6	0.3
③-5	Silty clay	31.3	5.09	26.4	16.0	0.3	7.2	0.3
③-7	Clay	38.6	3.60	21.4	16.9	0.35	10.0	0.3
–	Bridge pile-pulled area	–	–	–	–	–	–	–

### Identification effect analysis

We redefined the geological types based on geological exploration data of similar projects in the Hangzhou area before engineering application. The results of the redefined stratum are shown in Table 10. The geological types of the excavation face are classified into types based on the redefined stratum (Table 11). The model is then trained online using historical engineering data similar to the target project.

**Table 10:** Stratum redefinition results of the tunnel project from Kehua Road to the middle ventilating shaft.

Redefined stratum	Ordinary stratum number	Ordinary stratum name
1	③-1	Silt clay
	③-2	Muddy silty clay
2	③-7	Clay
	③-4	Clayey silt
	③-5	Silty clay
3	Bridge pile-pulled area	Bridge pile-pulled area

**Table 11:** Geological type redefinition result of the tunnel project from Kehua Road to the middle ventilating shaft.

Geological type	Type 1	Type 2	Type 3	Type 4
Redefined stratum included	1	1, 2	2	3

**Figure 9:**
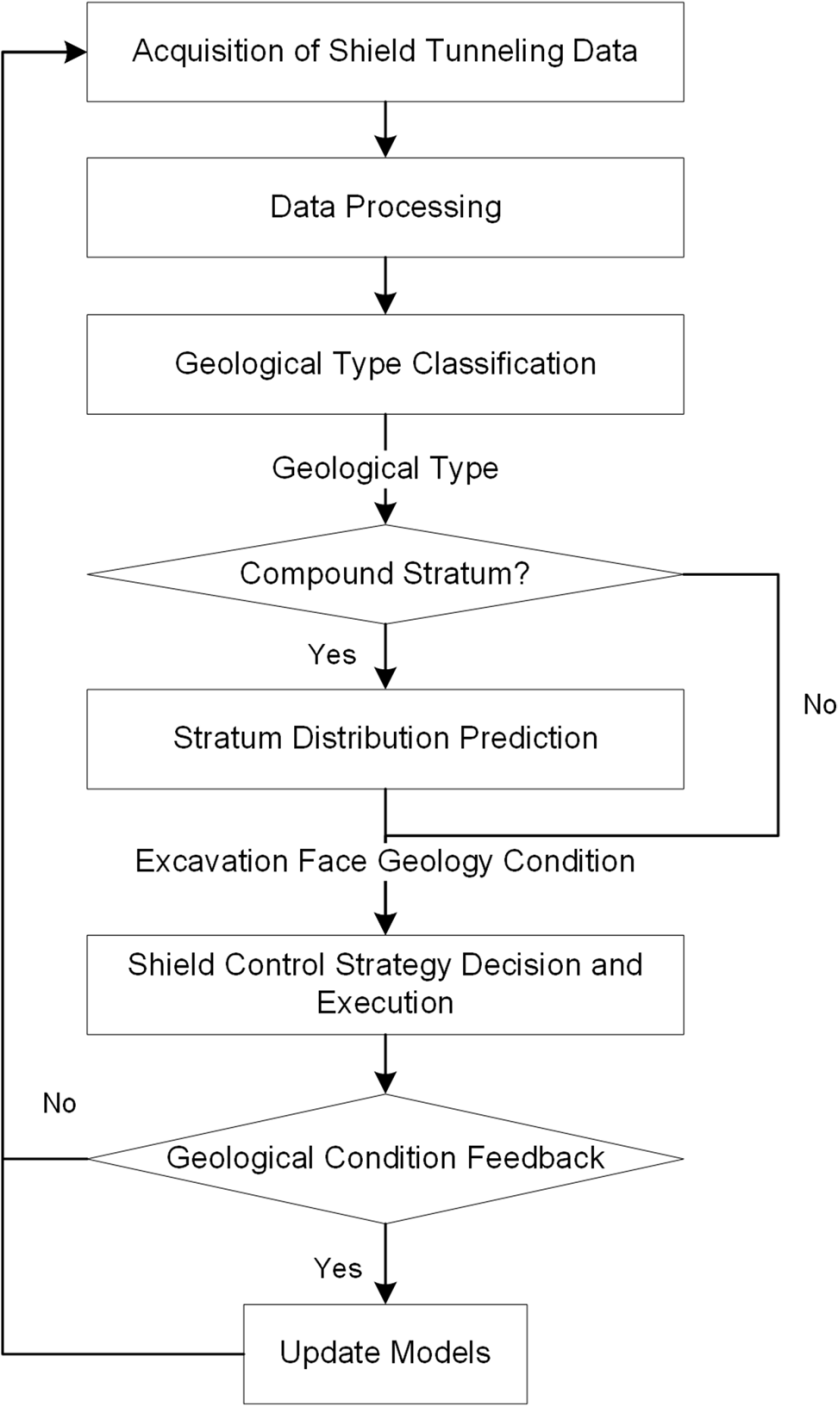
RGFI model application flow chart.

On this basis, real-time geological identification results are provided for the automatic tunnelling process based on real-time tunnelling data. The flow chart is shown in Fig. 9. Real-time prediction of geological conditions using RGFI during shield tunnelling and online updating of the models based on borehole data and feedback from shield operators. The model is consistent with the geological survey results in most cases, but in some sections, it differs significantly from the geological exploration results. The following is an analysis of the RGFI prediction results around the two cases of inconsistency with the results of geological exploration reports.

#### Tunnelling through an area with bridge piles

**Figure 10:**
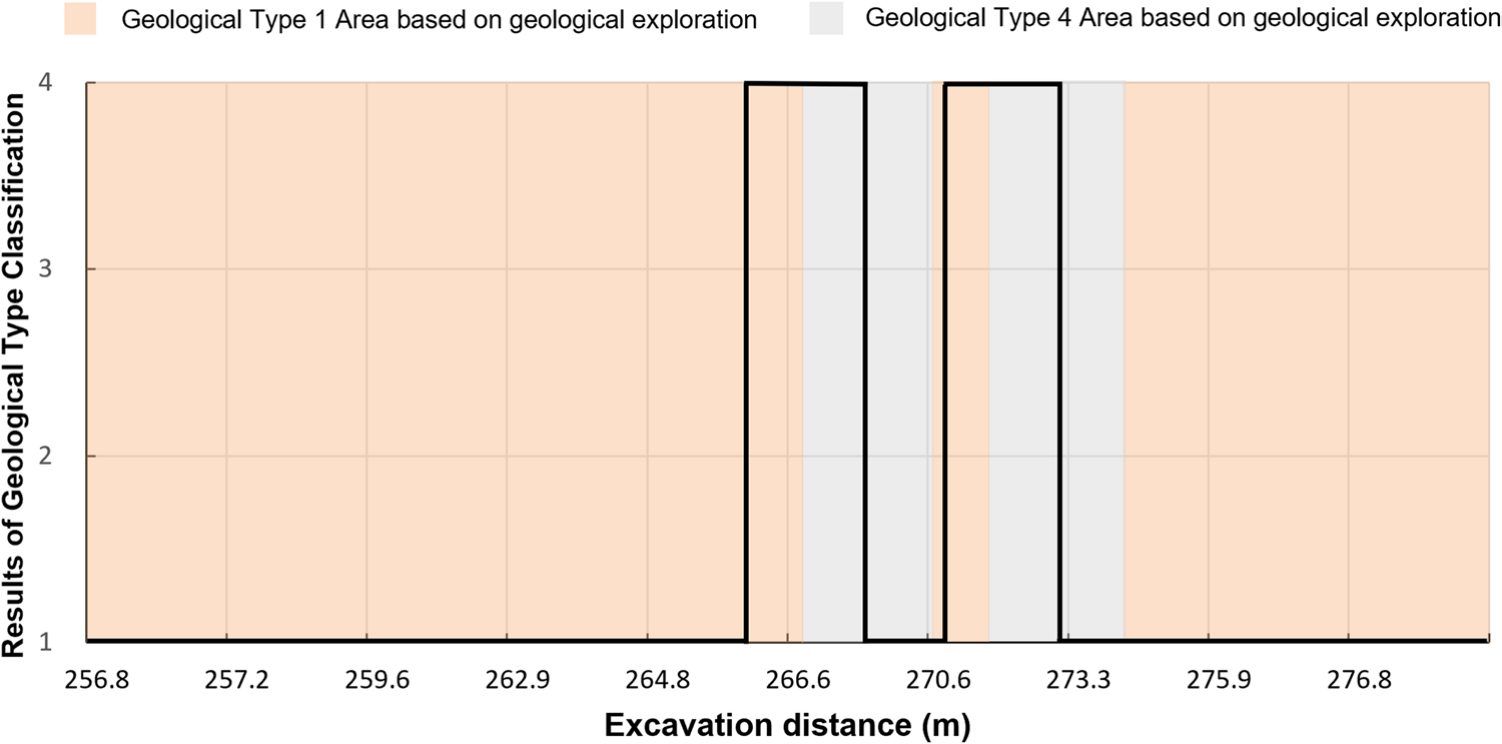
Geological type identification results from mileage 265–274 m.

According to geological exploration, there are two bridge piles located between mileage 265 m and 274 m before. The old piles foundation is buried deep, and the exact location is unknown, so taking countermeasures prematurely will affect the construction progress. At the same time, untimely detection may lead to equipment safety problems. During the shield tunnelling, the RGFI identifies the geological conditions in real-time. Figure 10 shows the identification result. The orange and gray parts in the figure are the soft soil area and bridge pile location based on geological exploration, respectively. The black line is the result of geological type classification. The geological exploration results and the model results do not fully match. RGFI identifies the location of the bridge piles earlier.

**Figure 11:**
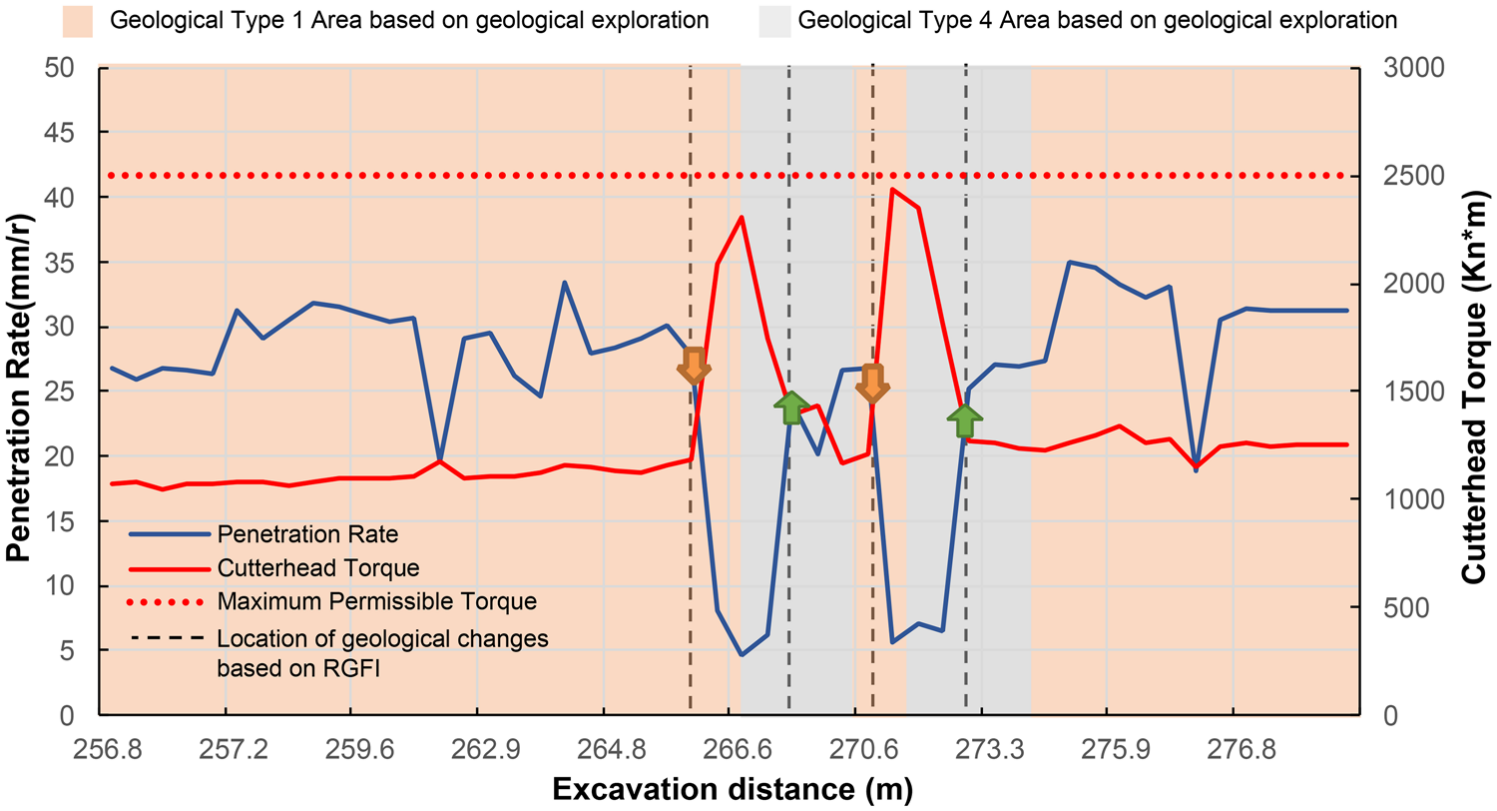
Cutterhead torque and penetration rate from mileage 265–274 m.

Then, the result of RGFI was fed back to the control strategy decision model of the self-driving shield, and the tunnelling parameters were adjusted. The adjustment of the tunnelling parameters was carried out by the control model in the position of the arrows in Fig. 11. Reduce the penetration rate by increasing the cutterhead speed and reducing the advance speed to avoid equipment failure caused by excessive cutterhead torque when pile-pulling area is determined. When the result of geological type determination isn't pile-pulling area, the original advance speed is restored. The final control effect shows that the timely adjustment of the shield tunnelling strategy based on the model geological condition identification results effectively avoids excessive cutter torque.

#### Tunnelling through a stratum distribution variation area

Geological conditions would change from mileage 796–811 m according to the geological exploration results. Due to the sparse borehole, precise geological conditions can’t be known from geological exploration. However, the shield attitude control strategy needs to be adjusted according to different geological conditions^47^. Accurate geological condition information is important for shield control in this area.

**Figure 12:**
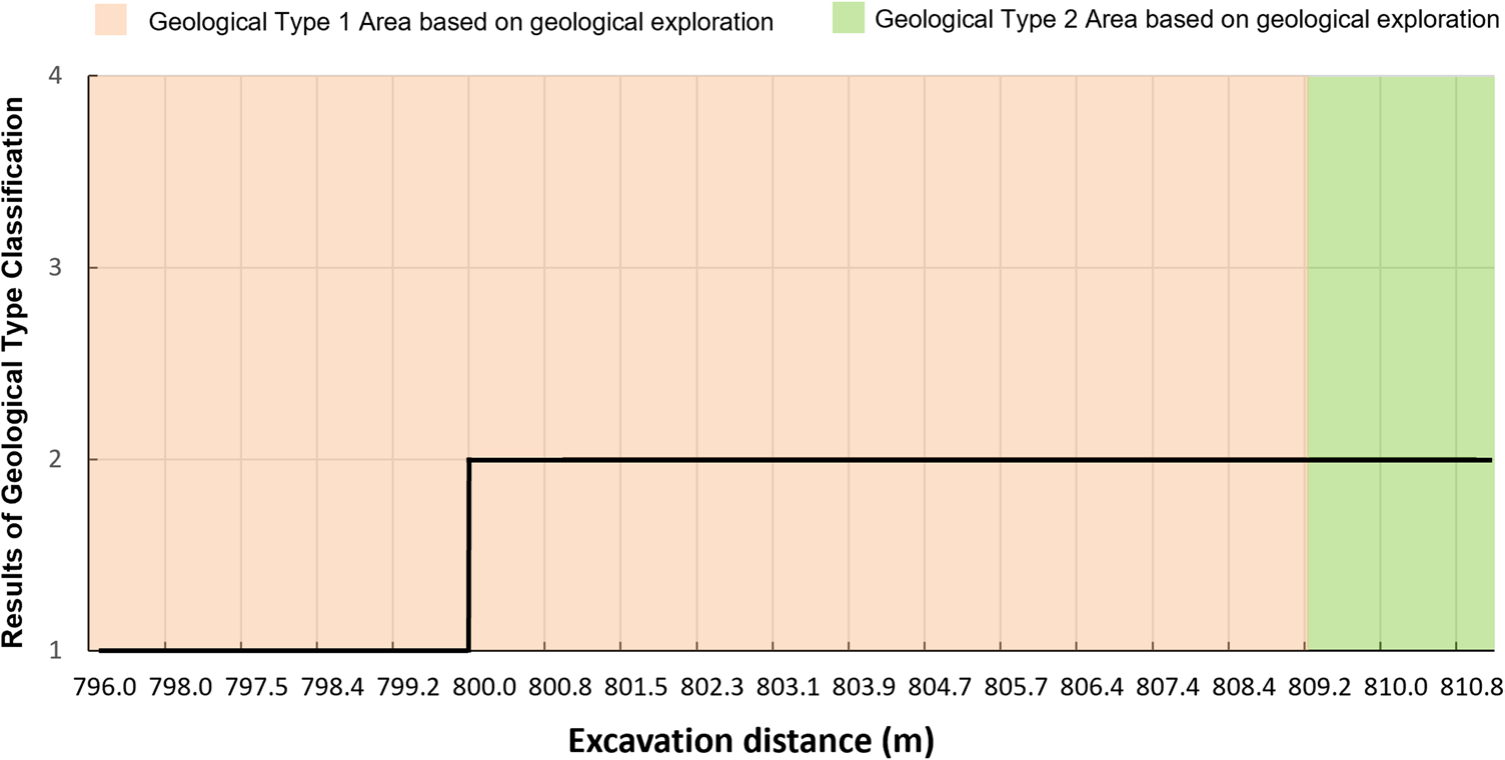
Geological type identification results from mileage 796–811 m.

**Figure 13:**
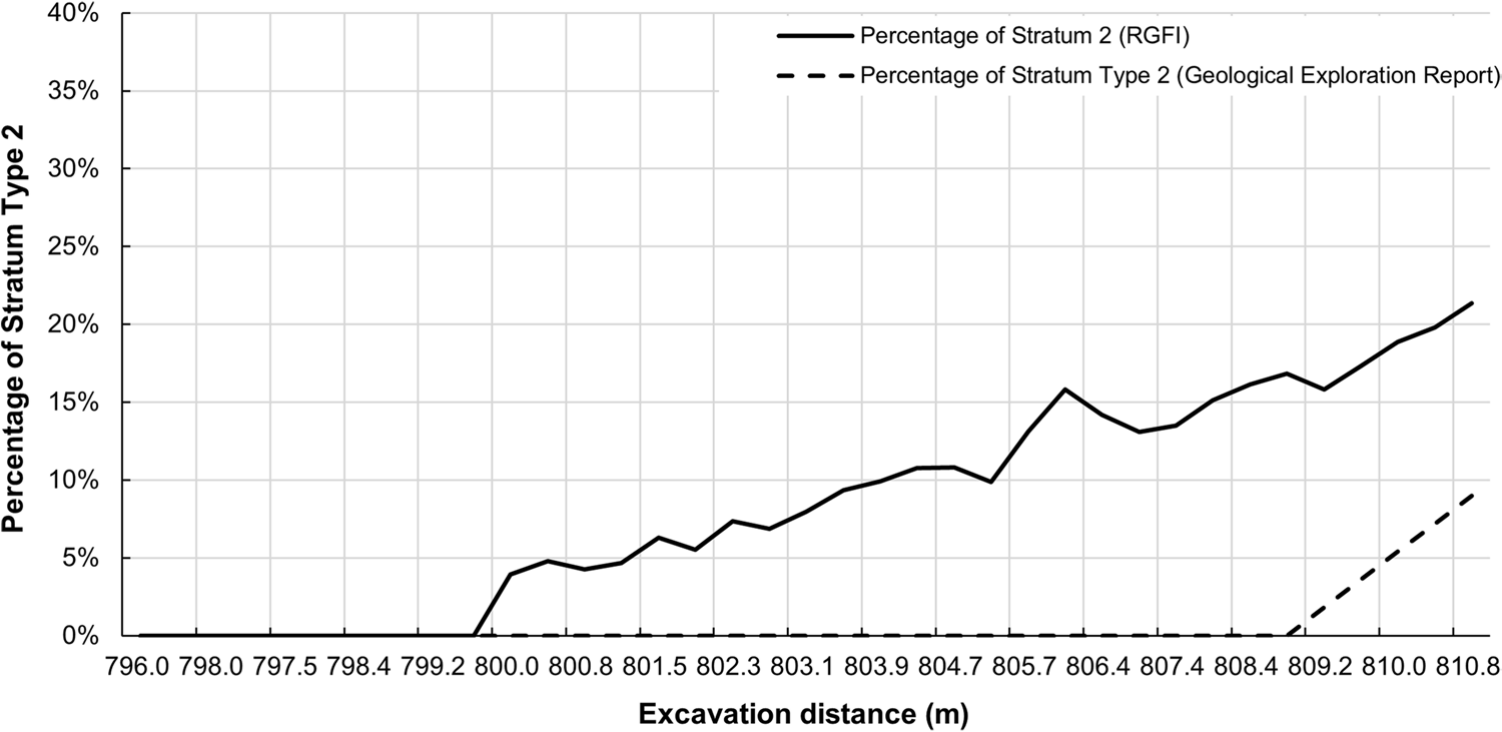
Stratum distribution prediction results from mileage 796–811 m.

**Figure 14:**
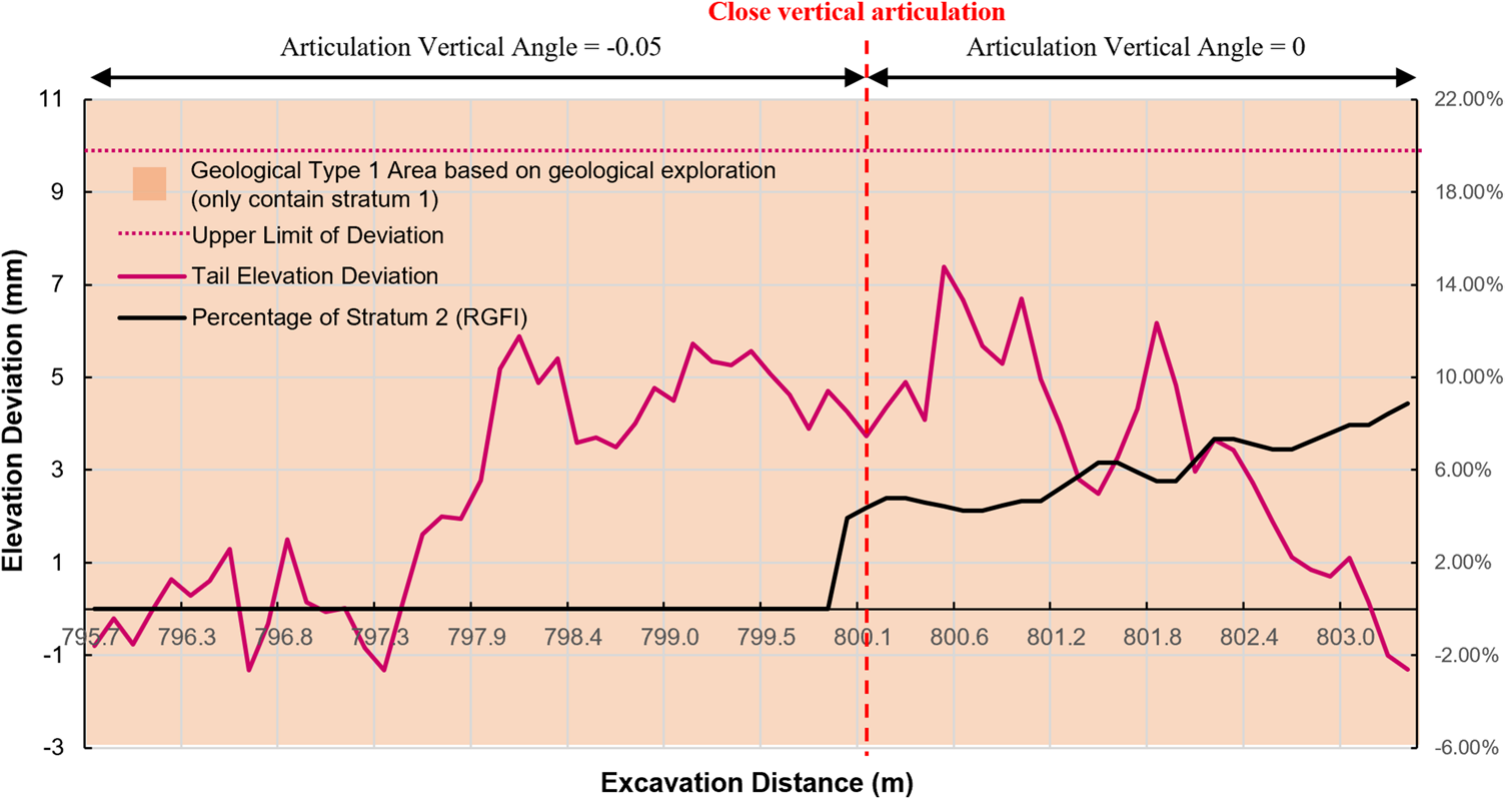
Diagram of shield control strategy adjustment from mileage 796–811 m.

From Fig. 12, it can be seen that the geological type classification model identifies a change from geological type 1 (containing stratum 1) to geological type 2 (containing stratum 1 and 2) at mileage 800 m. And according to the geological distribution model results (Fig. 13), the percentage of stratum 2 rises rapidly after 800 m, which is different from the geological exploration report. Based on the prediction results and the trend of shield tail deviation, the shield articulation angle was adjusted from $-0.05^\circ$ to $0^\circ$ at mileage 800.2 m, as shown in Fig. 14. After the strategy adjustment, the shield tail deviation gradually returned to the ideal control position, avoiding the quality problems caused by excessive attitude deviation. Both the feedback of shield tunnelling parameters and the shield control effect show that the geological condition judgment given by RGFI is accurate.

## Conclusions

In this study, the Rapidly Geological Features Identification (RGFI) method is proposed, which uses multistage machine learning methods to improve the model accuracy while ensuring the model's generalization performance and provides strong support for the adjustment of the shield control strategy. This method has three advantages. First, we redefine the stratum based on the geotechnical parameters related to the shield tunnelling height, which reduces the number of classifications and improves the guidance for shield construction. Second, the precise geological condition prediction was divided into two stages. Classification of the stratigraphic combination is performed first and then predicts the stratum distribution. This approach improves the generalization ability of the model. Third, the important differences in tunnelling parameter features under different stratigraphic combinations are considered using the attention mechanism. A deep neural network with an attention mechanism effectively improves the accuracy of stratum distribution prediction.

The RGFI method has shown better accuracy than other methods in engineering simulation experiments. In the Hangzhou-Shaoxing tunnel project, the RGFI model achieved good results and successfully supplemented or corrected the geological descriptions that were not detailed or precise in the geological exploration report, providing accurate geological information for self-driving shield decision-making and ensuring the quality and safety of construction.

Admittedly, the RGFI method requires a large amount of construction data under similar geological conditions for model training, so its application has some limitations. For example, when the shield faces a new geological environment, the model may misjudge the situation, posing a particular risk to the self-driving shield. Thus, in subsequent research, we will look for a method that can adapt to unknown geological conditions, use known geological types and characteristics to refine the description of unknown geology and provide more effective technical support for shield tunnelling.

## Data Availability

The datasets generated during and/or analyzed during the current study are not publicly available but are available from the corresponding author on reasonable request.

## References

[1] Hu M (2022). Self-driving shield: Intelligent systems, methodologies, and practice. Autom. Constr..

[2] Bouayad D, Emeriault F (2017). Modeling the relationship between ground surface settlements induced by shield tunneling and the operational and geological parameters based on the hybrid PCA/ANFIS method. Tunn. Undergr. Space Technol..

[3] Ji J (2021). An efficient probabilistic design approach for tunnel face stability by inverse reliability analysis. Geosci. Front..

[4] Jihua Y, Changbin Y, Dong M, Yao W (2019). comprehensive advanced geological prediction methods for tunnel construction with double sheild Tbm. J. Eng. Geol..

[5] Yubo Li (2017). Application of 3-D seismic wave advanced geological forecast to TBM construction of Hanjiang-to-Weihe River Valley Water Diversion Project. Water Resour. Hydropower Eng..

[6] Kaus A, Boening W (2008). BEAM—Geoelectrical ahead monitoring for TBM-drives. Geomech. Tunn..

[7] Hoshino, T., Matsubara, K., Ozawa, Y. & Tanaka, Y. Development of Learning System for Shield Machine Atitude for Automatic Operation of Shield Machine. in VI–554 (2020).

[8] Cangsong LI, Ting GU, Ding J, Weigang YU, Faliang HE (2008). Horizontal sound probing (HSP) geology prediction method appropriated to TBM construction. J. Eng. Geol..

[9] Nelson, P., Orourke, T. & Kulhawy, F. H. Factors affecting TBM penetration rates in sedimentary rocks. in (1983). 10.1016/0148-9062(84)91489-x.

[10] Hassanpour J, Rostami J, Khamehchiyan M, Bruland A, Tavakoli HR (2010). TBM performance analysis in pyroclastic rocks: A case history of Karaj water conveyance tunnel. Rock Mech. Rock Eng..

[11] Leu, S.-S., Joko, T. & Sutanto, A. Applied real-time Bayesian analysis in forecasting tunnel geological conditions. In *2010 IEEE International Conference on Industrial Engineering and Engineering Management* 1505–1508 (2010). 10.1109/IEEM.2010.5674155.

[12] Guan Z, Deng T, Du S, Li B, Jiang Y (2012). Markovian geology prediction approach and its application in mountain tunnels. Tunn. Undergr. Space Technol..

[13] Ioannou PG (1987). Geologic prediction model for tunneling. J. Constr. Eng. Manag..

[14] Miranda T, Gomes Correia A, Ribeiro e Sousa L (2009). Bayesian methodology for updating geomechanical parameters and uncertainty quantification. Int. J. Rock Mech. Min. Sci..

[15] Zhu B, Guofang G, Rulin Z, Guobin L (2011). Identification of strata with BP neural network based on parameters of shield driving. J. Zhejiang Univ. Sci..

[16] Zhang W (2020). State-of-the-art review of soft computing applications in underground excavations. Geosci. Front..

[17] Zhang Q, Yang K, Wang L, Zhou S (2020). Geological type recognition by machine learning on in-situ data of EPB tunnel boring machines. Math. Probl. Eng..

[18] Shi, M. *et al*. Geology prediction based on operation data of TBM: comparison between deep neural network and soft computing methods. In *2019 1st International Conference on Industrial Artificial Intelligence* 1–5 (2019). doi:10.1109/ICIAI.2019.8850794.

[19] Liu Z, Li L, Fang X, Qi W, Zhang Y (2021). Hard-rock tunnel lithology prediction with TBM construction big data using a global-attention-mechanism-based LSTM network. Autom. Constr..

[20] Liu B (2019). Improved support vector regression models for predicting rock mass parameters using tunnel boring machine driving data. Tunn. Undergr. Space Technol..

[21] Liu B (2020). Prediction of rock mass parameters in the TBM tunnel based on BP neural network integrated simulated annealing algorithm. Tunn. Undergr. Space Technol..

[22] Jung J-H, Chung H, Kwon Y-S, Lee I-M (2019). An ANN to predict ground condition ahead of tunnel face using TBM operational data. KSCE J. Civ. Eng..

[23] Sebbeh-Newton S (2021). Towards TBM automation: on-the-fly characterization and classification of ground conditions ahead of a TBM using data-driven approach. Appl. Sci..

[24] Shinji, M., Akagi, W., Shiroma, H., Yamada, A. & Nakagawa, K. JH method of rock mass classification for tunnelling. in (OnePetro, 2002).

[25] Zhang Q, Liu Z, Tan J (2019). Prediction of geological conditions for a tunnel boring machine using big operational data. Autom. Constr..

[26] Yan T, Shen S-L, Zhou A, Chen X (2022). Prediction of geological characteristics from shield operational parameters by integrating grid search and K-fold cross validation into stacking classification algorithm. J. Rock Mech. Geotech. Eng..

[27] Yan T, Shen S-L, Zhou A (2022). Identification of geological characteristics from construction parameters during shield tunnelling. Acta Geotech..

[28] Guo Z (2021). A novel hybrid method for flight departure delay prediction using Random Forest Regression and Maximal Information Coefficient. Aerosp. Sci. Technol..

[29] Kezhi S, Shuxian S, Dajun Y, Mengshu W (2008). Fuzzy recognition for rock cuttability based on shield driving parameters. Chin. J. Rock Mech. Eng..

[30] Yu H (2021). Rock mass type prediction for tunnel boring machine using a novel semi-supervised method. Measurement.

[31] Niksefat N, Sepehri N (2001). Designing robust force control of hydraulic actuators despite system and environmental uncertainties. IEEE Control Syst. Mag..

[32] Helin Fu, Huangshi D, Zhen H, Xinyi C, Yunya Z (2020). Limit analysis of the thrust of the shield on the face passing underneath the sand layer. J. Railw. Eng. Soc..

[33] Hongxin W (2009). Effect of cutterhead compressing the front soil and influence of head aperture ratio on contact pressure of EPB shield to the front soil. China Civ. Eng. J..

[34] Zichang S, Shoujiu Li, Wei S, Maotian L, Chengang K (2011). Comparison analysis for computing earth pressure acted on excavation face in shield tunneling. J. Harbin Inst. Technol..

[35] Chinrungrueng C, Sequin CH (1995). Optimal adaptive k-means algorithm with dynamic adjustment of learning rate. IEEE Trans. Neural Netw..

[36] Umargono, E., Suseno, J. E. & S. K., V. G. K-Means Clustering Optimization using the Elbow Method and Early Centroid Determination Based-on Mean and Median. In *Proceedings of the International Conferences on Information System and Technology* 234–240 (SCITEPRESS - Science and Technology Publications, 2019). 10.5220/0009908402340240.

[37] Marutho, D., Handaka, S., Wijaya, E., & Muljono. The Determination of Cluster Number at k-Mean Using Elbow Method and Purity Evaluation on Headline News. 538 (2018). 10.1109/ISEMANTIC.2018.8549751.

[38] Memon, N., Patel, S. B. & Patel, D. P. Comparative Analysis of Artificial Neural Network and XGBoost Algorithm for PolSAR Image Classification. In *Pattern Recognition and Machine Intelligence* (eds. Deka, B. et al.) 452–460 (Springer International Publishing, 2019). 10.1007/978-3-030-34869-4_49.

[39] Song K, Yan F, Ding T, Gao L, Lu S (2020). A steel property optimization model based on the XGBoost algorithm and improved PSO. Comput. Mater. Sci..

[40] Zhou Q, Zhou H, Zhou Q, Yang F, Luo L (2014). Structure damage detection based on random forest recursive feature elimination. Mech. Syst. Signal Process..

[41] Zou F (2021). An anti-noise one-dimension convolutional neural network learning model applying on bearing fault diagnosis. Measurement.

[42] Shen S-L, Elbaz K, Shaban WM, Zhou A (2022). Real-time prediction of shield moving trajectory during tunnelling. Acta Geotech..

[43] Elbaz K, Shen S-L, Zhou A, Yin Z-Y, Lyu H-M (2021). Prediction of disc cutter life during shield tunneling with AI via the incorporation of a genetic algorithm into a GMDH-type neural network. Engineering.

[44] Xu Z, Li C, Yang Y (2021). Fault diagnosis of rolling bearings using an improved multi-scale convolutional neural network with feature attention mechanism. ISA Trans..

[45] Caruana, R. & Niculescu-Mizil, A. An Empirical Comparison of Supervised Learning Algorithms. In *Proceedings of the 23rd International Conference on Machine Learning* 161–168 (Association for Computing Machinery, 2006). 10.1145/1143844.1143865.

[46] Dahl, G. E., Sainath, T. N. & Hinton, G. E. Improving deep neural networks for LVCSR using rectified linear units and dropout. In *2013 IEEE International Conference on Acoustics, Speech and Signal Processing* 8609–8613 (2013). 10.1109/ICASSP.2013.6639346.

[47] Hu M, Zhang H, Wu B, Li G, Zhou L (2022). Interpretable predictive model for shield attitude control performance based on XGboost and SHAP. Sci. Rep..

